# Predictors of successful emergency shock wave lithotripsy for acute renal colic

**DOI:** 10.1007/s00240-022-01332-3

**Published:** 2022-06-03

**Authors:** Adel Kurkar, Ahmad A. Elderwy, Mahmoud M. Osman, Islam F. Abdelkawi, Mahmoud M. Shalaby, Mohamed F. Abdelhafez

**Affiliations:** Department of Urology, Urology and Nephrology Hospital, Faculty of Medicine, Assiut University, Assiut, 71515 Egypt

**Keywords:** Emergency shockwave lithotripsy, Renal colic, Ureteral stone

## Abstract

The role of emergency shockwave lithotripsy (SWL) in persistent pain control in patients with ureteral stones is not well established. The aim of this study is to evaluate efficacy as well as the predicting variables for successful early SWL patients with symptomatic ureteral stones. Eighty-six patients with a persistent renal colic secondary to single ureteral stone (6–12 mm) were prospectively enrolled in this study. SWL was performed within 24 h of the onset of flank pain. Pain control and stone-free rate after emergency SWL session were 58.1% and 44.2%, respectively. Seven patients required post-SWL ureteroscopy and ureteral stent placement for uncontrolled pain. The overall 3-month stone-free rate after SWL monotherapy was 83.7%. On multivariate analysis, predictors for pain relief after emergency SWL were lower Hounsfield (HU) stone density, mild hydronephrosis (HN) at presentation and presentation during the first colic episode. Lower HU stone density was the single predictor of successful stone clearance after single emergency SWL session on multivariate analysis. In conclusion, early SWL is feasible and effective in management of ureteral stones presented by renal colic with low HU.

## Introduction

The prevalence of nephrolithiasis is reported to be 13% [1]. In addition, there is also a noticeable increase in the number of patients who receive an active treatment for upper urinary tract calculi (UUTC), Park et al., (2016) reported an increase of different modalities of intervention including SWL, ureteroscopy (URS) and percutaneous nephrolithotomy (PNL) by 102, 110, and 180%, respectively, over the last 10 years [2]. Although many factors are expected to influence the treatment decision for patients with UUTC, pain relief remains the first concern in an acute episode [3]. Non-steroidal anti-inflammatory drugs (NSAID) are the first-line drugs with a better efficacy in pain control compared to opioids only or combined opioids and antispasmodics [4]. Pethidine in particular should be avoided because of the higher rate of vomiting and need for further analgesia [5].

Traditionally, after control of pain, the definitive treatment of stones was delayed with options including medical expulsive therapy (MET), SWL, and ureteroscopy [6]. Recently, the utilization of both SWL and URS as emergency procedures with the aim to decrease the time of stone-related symptoms, morbidity, and possible complications was found to be safe and effective [7].

In this study, we evaluate the efficacy as well as the predicting factors for successful emergency SWL in patients with persistent flank pain despite of receiving medical treatment.

## Patients and methods

After the approval of our institutional ethical review board, we conducted a prospective study including adults presented to emergency department of our hospital between July 2008 and June 2011 for acute renal colic. Persistent flank pain was defined as uncontrolled pain after receiving 2 doses of 30 mg of intravenous Ketorolac within 6 h. Inclusion criteria were patients who are 18 year old or older with persistent flank pain secondary to medium sized single ureteral stone (6–12 mm).

We excluded from this study patients with history of surgical intervention, patients with evidence of urinary tract infection and or renal failure, patients with stone size > 12 mm or less than 6 mm, marked hydronephrosis or peri-renal urinoma, patients with ureteral stricture and patients with solitary kidney or bilateral hydronephrosis.

Sample size calculation was carried out using Epi-info™, version 3.3 (CDC, 2005). A calculated sample of 73 or more was needed, with a *p* value < 0.05 and 95% power.

A total of 163 patients were assessed for eligibility. Monitoring of vital signs, plain X-ray of the urinary tract (KUB), abdominal ultrasound, non-contrast computed tomography (NCCT) of the abdomen and pelvis, urinalysis, complete blood count, and coagulation profile were done for all patients.

Characterization of the stone was based on the KUB and NCCT imaging and included stone size (largest longitudinal and transversal diameter measured by CT), stone location (lumbar, iliac or pelvic), stone outline (smooth or irregular), and stone density measured by Hounsfield units (HU). Radiography with KUB was performed in all patients to assess whether the stone was radio-opaque or not. The presence of hydronephrosis was defined by ultrasound and patients were divided into 3 groups according to the degree of HN. Mild HN if the renal pelvis only was dilated, moderate HN if the renal pelvis and some calices were dilated, and severe HN if had severe uniform dilatation of the renal pelvis and calices with cortical thinning.

Based on our inclusion and exclusion criteria, 86 patients (52 men, 34 women) were included in the study and a written consent was signed. They underwent emergency extracorporeal shock wave lithotripsy (SWL) as soon as possible (within 24 h of the onset of colicky pain) using a Dornier Lithotripter S with dual ultrasound/fluoroscopic monitoring. All patients received intravenous perfusion of 500 ml saline, 1 gm of IV first-generation cephalosporin and 30 mg ketorolac IV 20 min before the session. No anesthesia was used, but 0.1 mg/kg diluted IV morphine was given during the session.

Lumbar ureteral stones were fragmented with the patient in the supine position, iliac and pelvic stones in prone position. SWL protocol with a total count of 4000 SW/session at power of 16–20 kV and frequency of 60–70/min was utilized.

The primary end point after the emergency SWL was pain relief (VPAS ≤ 3) after the SWL session and the secondary end point was stone clearance which was checked with ultrasound and KUB at the end of session or CT KUB if needed, as well as 3 weeks later.

Statistical analysis was performed using inter-cooled STATA®, (version 9.2). A univariate analysis was done to compare the two treatment groups. Analysis included the Chi-square test or Fisher’s exact test for comparison of the categorical data, and the Mann–Whitney *U* test compare the non-categorical data. A multiple regression model was used for factors maintaining a statistically significant impact on the time to stone clearance, indicating that they act independently.

## Result

Eighty-six patients were enrolled; the basic characteristics of patients at presentation are summarized in Table 1.

**Table 1:** The basic characteristics of patients underwent emergency SWL

Characteristics	*N* (%)
Age (mean ± SD)	39.9 ± 14.1
Sex (M:F)	(17:19)
Stone diameter mm (mean ± SD)	8.8 ± 1.3
Stone outline smooth: irregular	38:48
HFU (mean ± SD)	842.7 ± 1232.1
BMI (mean ± SD)	23.9 ± 2.7
Pain relief	50 (58%)
Single session success	38 (44.2%)
Presence of hydronephrosis	55 (63.9)
Laterality (L:R)	39:47
Stone location	
Upper	46
Middle	12
Lower	28

*HFU* (Hounsfield unit), *BMI* (Body mass index)

**Fig. 1:**
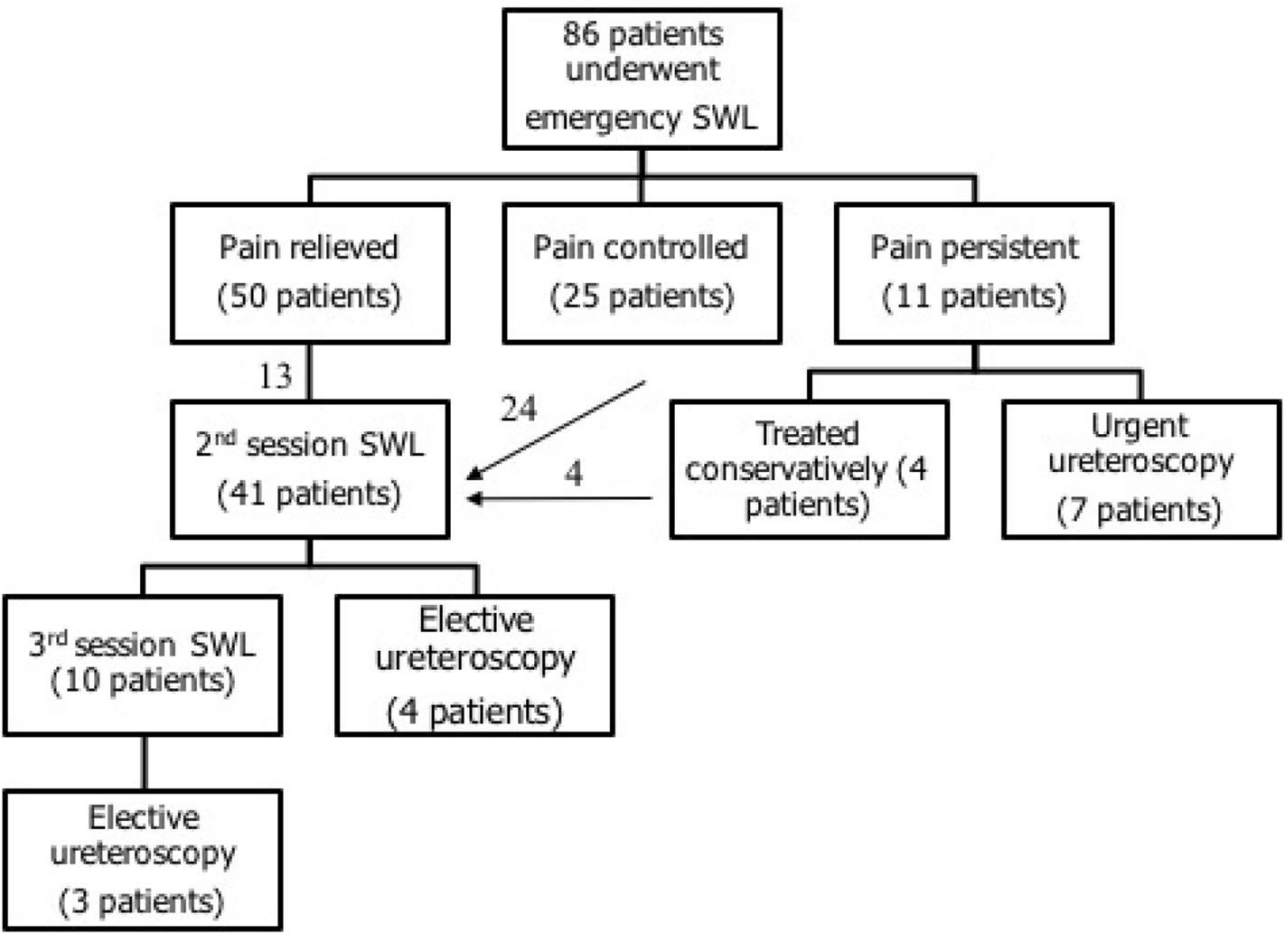
Step by step management of stone ureter in patients with renal colic

Pain relief (VPAS ≤ 3) and stone-free rate after emergency SWL session were 58.1% and 44.2%, respectively. Refractory pain (VPAS ≥ 7) occurred in 11 patients (12.7%); of them 7 required urgent ureteroscopy. The remaining 25 patients (29%) reported a controlled (tolerable) pain with VPAS (4–6) and of them 24 required a second SWL session (Fig. 1).

The overall 3-month stone-free rate after SWL monotherapy (up to 3 sessions) was 83.7%. Ureteroscopy was used in (7, 4, and 3) patients after first, second and third SWL sessions, respectively, i.e., a total of 14 patients (16.2%).

On multivariate analysis, predictors for pain relief after emergency SWL were lower HU stone density (OR 0.992, 95% CI 0.985–0.998, *p* = 0.016), mild HN at presentation (OR 9.904, 95% CI 1.058–92.689, *p* = 0.044) and presentation during the first colic episode (OR 5.345, 95% CI 1.036–27.573, *p* = 0.045).

Those with relieved renal pain after early SWL had median HU stone density of 650 compared to 990 for those with persistent renal pain (*p* < 0.001) (Table 2). Lower HU stone density was also the single predictor of successful stone clearance after single emergency SWL session on multivariate analysis (OR 0.978, 95% CI 0.963–0.993, *p* = 0.004) (Table 3).

**Table 2:** Predictors for pain relief after emergency SWL (multivariate analysis)

Variable	Pain relief after emergency SWL	
	OR (95% CI)	*P* value
Age	1.00 (0.94–1.06)	0.913
Male	4.05 (0.67–24.47)	0.128
BMI	0.80 (0.50–1.27)	0.339
Presentation during the first colic episode	5.35 (1.04–27.57)	0.045*
Stone at lumbar ureter	0.90 (0.18–4.41)	0.899
Stone length	0.34 (0.06–1.79)	0.075
Stone width	1.30 (0.30–5.60)	0.160
Irregular stone outline	2.20 (0.44–10.96)	0.641
HU stone density	0.992 (0.985–0.998)	0.016*
Mild HN at presentation	9.90 (1.06–92.69)	0.044*

*p < 0.05

**Table 3:** Predictors for stone clearance after emergency SWL (multivariate analysis)

Variable	Stone clearance after emergency SWL	
	OR (95% CI)	*P* value
Age	0.99 (0.90–1.09)	0.822
Male	1.20 (0.07–20.28)	0.901
BMI	1.02 (0.52–1.98)	0.961
Presentation during the first colic episode	1.77 (0.18–17.05)	0.623
Stone at lumbar ureter	0.89 (0.07–10.92)	0.927
Stone length	0.80 (0.26–2.44)	0.690
Stone width	1.03 (0.08–12.73)	0.983
Irregular stone outline	0.49 (0.03–7.59)	0.610
HU stone density	0.98 (0.96–0.99)	0.004*
Mild HN at presentation	1.98 (0.13–29.72)	0.622

## Discussion

Despite of the well-established role of SWL as a minimally invasive tool for the management of ureteral stones, there has been no consensus about its role in the emergency setting especially in those with refractory renal colic. Nowadays with the revolutionary improvements of SWL machines and their readily availability such a role has to be legitimately established [8].

Many factors affect the choice of the modality of ureteral stone treatment. For ureteral stones > 6 mm, both URS and SWL have higher stone-free rate compared to medical expulsive therapy. Both modalities will render the patient more rapidly stone free without the prolonged use of multiple medications and minimize trips to the emergency department for pain control [9].

The balance between SWL and URS is not that simple. With an overall stone-free rate of 90%, URS may exemplify an over treatment considering that the patient will undergo a surgical procedure under anesthesia with the possible complications of both [5]. Since its first introduction [10], it has been previously shown that immediate SWL is a safe procedure and have good outcomes in the first 24 h, moreover they reduce the requirement of auxiliary procedures and the need of hospitalization [11–13].

In this study, emergency SWL was effective in pain control in 58.1% of patients. The predictor for success pain control was low HU stone density. In addition, 44.2% of patients were stone free after emergency SWL session. Predictors for stone clearance were lower HU stone density, mild HN at presentation and presentation during the first colic episode.

Panah A et al., found that stone size and Hounsfield units are important factors that affect the success of the emergency SWL, this was in agreement with our study which found that Lower HU stone density was also the single predictor of successful stone clearance after single emergency SWL session on multivariate analysis [14].

Cornelius et al. showed different results when they compared success rate of early SWL versus delayed SWL and found that BMI > 30 is the predictor of stone-free rate on multivariate analysis [15]. While Ghaliani et al. concluded that stone size may be the main predictive factor for successful emergency SWL [16]. In fact, we did not find that age, sex, BMI, stone length, stone location, stone outline or hydronephrosis affect stone clearance in our study.

To the best of our knowledge, this study is the first one to investigate the predictors of successful SWL and pain relief during the acute attack of renal colic. We found that lower HU stone density, mild HN at presentation and presentation during the first colic episode are predictors for pain relief.

## Conclusion

Emergency SWL is an effective modality combining both pain relief and the definitive treatment of ureteral stones. Our analysis indicate that patients who present during the first colic episode with mild backpressure changes and have low HU stone density are the most likely to benefit from this approach.

## Data Availability

All related data and materials are available from the corresponding author upon request.
